# The amelioration of cartilage degeneration by photo-crosslinked GelHA hydrogel and crizotinib encapsulated chitosan microspheres

**DOI:** 10.18632/oncotarget.15750

**Published:** 2017-02-27

**Authors:** Pengfei Chen, Sheng Mei, Chen Xia, Ren Zhu, Yichuan Pang, Jiying Wang, Jianfeng Zhang, Fangchun Shao, Shunwu Fan

**Affiliations:** ^1^ Department of Orthopaedics, Sir Run Run Shaw Hospital, School of Medicine, Zhejiang University, Hangzhou, 310016, China; ^2^ Key Laboratory of Biotherapy of Zhejiang Province, Hangzhou, 310016, China; ^3^ Department of Orthopaedics, Yiwu Chowzhou Hospital, Yiwu, 322000, China; ^4^ MOE Key Laboratory of Macromolecular Synthesis and Functionalization, Department of Polymer Science and Engineering, Zhejiang University, Hangzhou, 310016, China; ^5^ Department of Pulmonary, Zhejiang Provincial People's Hospital, Hangzhou, 310016, China

**Keywords:** osteoarthritis, hydrogel, angiogenesis

## Abstract

The present study aimed to investigate the synergistic therapeutic effect of decreasing cartilage angiogenesis via exposure to crizotinib encapsulated by chitosan microspheres and photo-crosslinked hydrogel, with the goal of evaluating crizotinib as a treatment for osteoarthritis. First, we developed and evaluated the characteristics of hydrogels and chitosan microspheres. Next, we measured the effect of crizotinib on the cartilage degeneration induced by interleukin-1β in chondrocytes. Crizotinib ameliorated the pathological changes induced by interleukin-1β via its anti-angiogenesis function. In addition, we surgically induced osteoarthritis in mice, which were then injected intra-articularly with crizotinib-loaded biomaterials. Cartilage matrix degradation, expression of vascular endothelial growth factor and extracellular signal-regulated kinases 1/2 were evaluated after surgery. Treatment with the combination of crizotinib-loaded biomaterials retarded the progression of surgically induced osteoarthritis. Crizotinib ameliorated cartilage matrix degradation by promoting anti-angiogenesis and impeding extracellular signal-regulated kinases 1/2 signaling pathway. Our results demonstrate that the combination of photo-crosslinked hydrogel and crizotinib-loaded chitosan microspheres might represent a promising strategy for osteoarthritis treatment.

## INTRODUCTION

Osteoarthritis (OA) represents one of the leading causes of disability in older people worldwide [1]. Patients with OA undergo changeable levels of cartilage degeneration [2]. Structure-modifying prescriptions show promise as valid medicinal agents in OA treatment and worth further study [3].

Vascular endothelial growth factor (VEGF) is known to be an important mediator of angiogenesis [4], whereas cartilage is a tissue without vascular [5]. However, recent investigations have shown that angiogenic factors including VEGF are involved in both physiologic and pathologic cartilage metabolism [6, 7]. The expression of VEGF is mediated by hypoxia or by various growth factors such as interleukin-1β (IL1β) or tumor necrosis factor-α (TNFα) [6]. In addition, treatment via intravenous administration of an anti-VEGF antibody notably decreases OA symptoms in animal models [8]. Thus, targeting VEGF in OA might be beneficial for human OA therapy.

Crizotinib is an inhibitor of the mesenchymal-epithelial transition (c-MET)/hepatocyte growth factor (HGF) receptor [9]. Recent studies have demonstrated that Met activation by HGF stimulates the production of VEGF and facilitates angiogenesis [10]. Furthermore, crizotinib was shown to reduce VEGF production *in vitro* and tumor vascularization *in vivo* [10–12]. Additionally, a recent clinical report indicated that crizotinib may exert therapeutic effects on OA symptoms [13]. These findings suggest that crizotinib might ameliorate OA symptoms via its antiangiogenic effect.

Gelatin methacrylate (GelMA) hydrogels have been widely used for various biomedical applications because of their many attractive properties in adhesion and thermo sensitivity [14, 15]. Furthermore, photo-crosslinking represents a cost-efficient, fast, and common method of manufacturing materials with stable properties [16]. Hyaluronic acid (HA), a sole non-sulfated glycosaminoglycan, is involved in cell proliferation, morphogenesis, inflammation, and wound repair [17]. Previous studies have shown that hyaluronic acid methacrylate (HAMA) is an effective hydrogel for medical utilization and can be applied for cartilage regeneration [18]. Notably, by photo-crosslinking GelMA and HAMA hydrogels (GelHA hydrogel), the hybrid material demonstrated significantly better cell proliferation and mechanical characteristics compared with HAMA or GelMA in prior analyses [19]. Chitosan microspheres (CMs) have been established as a useful tool for drug delivery, as they are easy to prepare and can release drugs stably, including in OA [20]. CMs have properties such as biological compatibility, non-allergenic, and biodegradability; as a result, CMs can successfully provide site-specific drug delivery [21].

In this study, we assessed the effect of combining the GelHA hydrogel and crizotinib-loaded CMs for OA treatment. Firstly, we fabricated and tested the properties of GelHA hydrogels and CMs. We further utilized crizotinib in OA animal models. We suggested that crizotinib-loaded CMs combined with GelHA hydrogel could effectively delay OA progression.

## RESULTS

### Preparation and characterization of GelHA hydrogels

**Figure 1:**
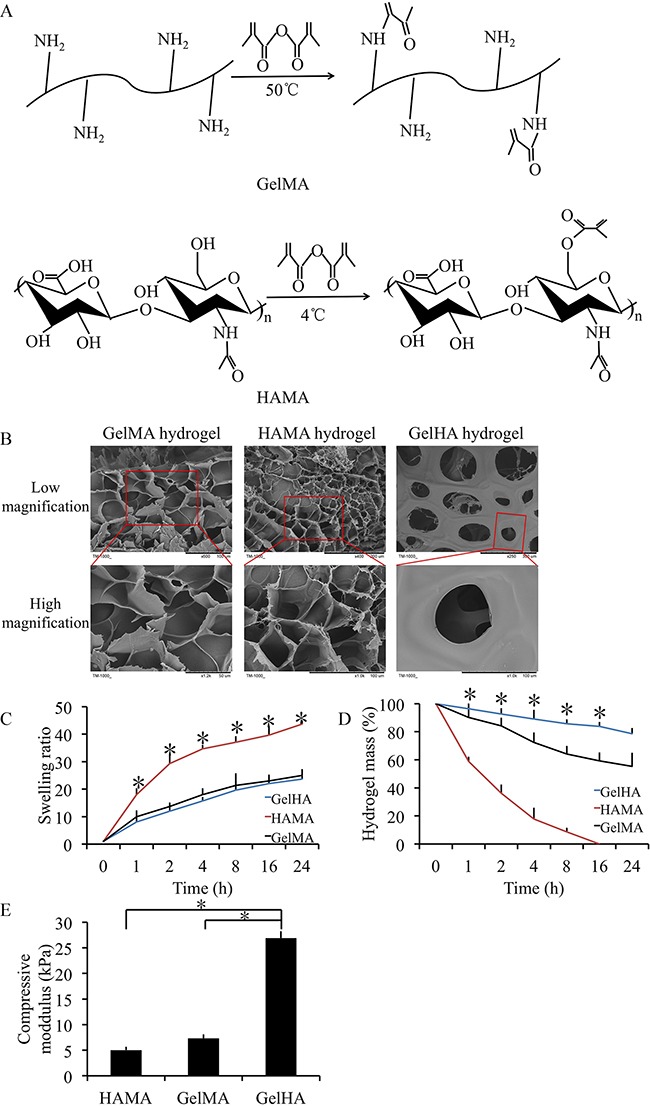
Preparation and characterization of GelHA hydrogels **(A)** Molecule structures and synthesis of polymer precursors of gelatin methacrylate (GelMA) and hyaluronic acid methacrylate (HAMA) hydrogels. **(B)** Microscopic structure of the hydrogels (low and high magnification). **(C)** Swelling kinetics of the hydrogels. n = 3, *p < 0.05 (one way ANOVA). **(D)** *In vitro* degradation profile of the hydrogels. n = 3, *p < 0.05 (one way ANOVA). **(E)** The compressive moduli for the hydrogels. n = 3, *p < 0.05 (one way ANOVA). GelHA hydrogel, hybrid hydrogel with the two components of GelMA and HAMA.

The GelHA hydrogel displayed a holey structure and stable pore size (50-100 μm, Figure 1B). Swelling and degradation testing was performed using different hydrogels. The swelling ratios were exhibited in Figure 1C. GelHA and GelMA hydrogels showed a significantly lower swelling ratio compared with HAMA hydrogels (p < 0.05) and there were no significant differences between GelHA and GelMA hydrogels (Figure 1C). In the hydrogel degradation tests, 21.4% of the GelHA hydrogels degraded in 24 h (Figure 1E). Compared with the HAMA and GelMA hydrogels, GelHA hydrogels showed a significant lower degradation rate (p < 0.05) (Figure 1D). Furthermore, the compressive modulus was 26.8±1.2 KPa for the GelHA hydrogels, which was significant higher than those for the HAMA and GelMA hydrogels (p < 0.05) (Figure 1E, Supplementary Figure 1).

### *In vitro* crizotinib release in chitosan microspheres

**Figure 2:**
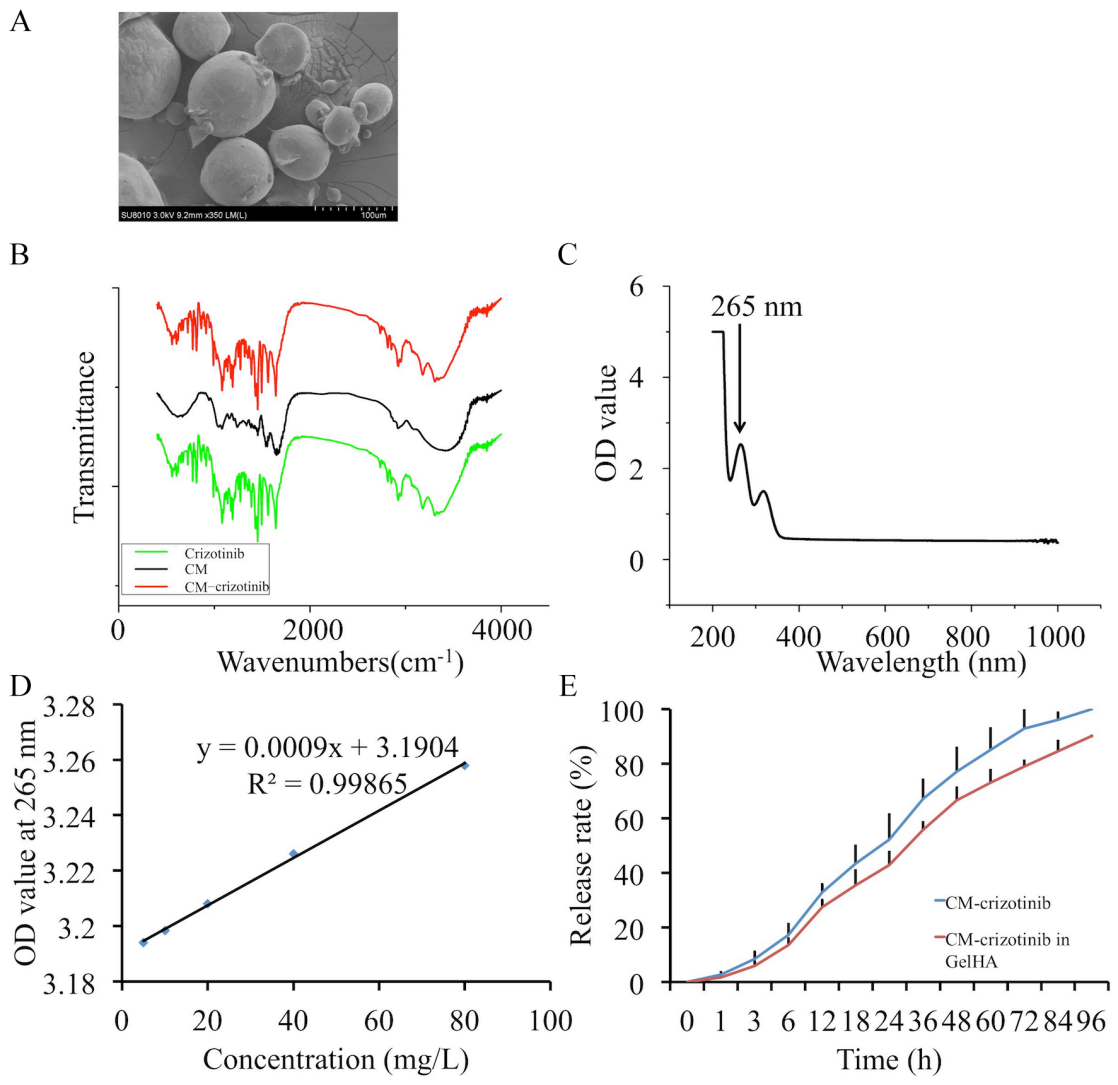
*In vitro* crizotinib release in chitosan microspheres **(A)** Scanning electron micrographs of crizotinib-loaded chitosan microspheres. **(B)** Fourier transform infrared (FTIR) spectroscopy for chitosan microspheres. **(C)** Absorption curve of crizotinib. **(D)** Standard curve of crizotinib. **(E)** Controlled release profile of chitosan microspheres.

The CMs displayed a spherical structure with a diameter of 40-100 μm (Figure 2A). Fourier transform infrared (FTIR) spectroscopy demonstrated that crizotinib was enclosed in CMs (Figure 2B). The absorption curve showed that the drug had absorption peaks at 265 and 317 nm (Figure 2C). The specification curve of crizotinib showed a fine linear relevance between 0–80 mg/L in PBS (Figure 2D). The crizotinib encapsulated in CM was completely released within 4 days and the release rate was high in the first day. The hydrogel had no significant influence on the release profile of crizotinib (Figure 2E).

### VEGF expression in degenerative cartilage

**Figure 3:**
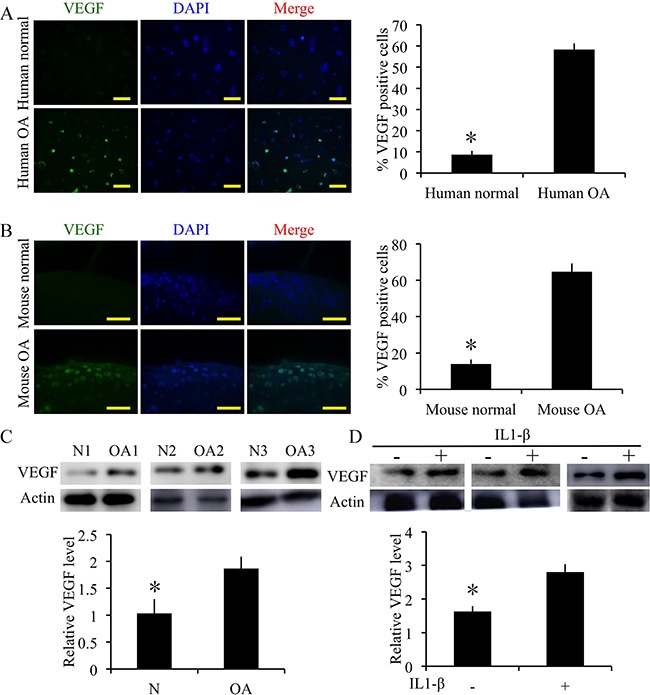
Expression of VEGF in cartilage samples from human patients with OA and mice with surgically induced OA **(A)** Immunofluorescence staining and quantification of VEGF-positive cells of VEGF in normal and OA human cartilage samples. Scale bars = 100 μm. **(B)** Immunofluorescence staining and quantification of VEGF-positive cells of VEGF in normal and OA mouse cartilage samples. Scale bars = 100 μm. n = 3, *p < 0.05 (Student's *t*-test). **(C)** Western blot analysis of VEGF level in human OA and normal cartilage (cropped blots are displayed). n = 3, *p < 0.05 (Student's *t*-test). **(D)** Western blot analysis of VEGF level when human OA chondrocytes treated with IL1β (10 ng/mL) and measurement of relative density of VEGF band (cropped blots are displayed). n = 3, *p < 0.05 (Student's *t*-test).

The expression of VEGF was detected by immunofluorescence or western blotting in degenerative cartilage. The degenerative cartilage exhibited significantly more up-regulated VEGF expressions compared to the normal human specimens (p < 0.05) (Figure 3A, 3C). Meanwhile, the degenerative mouse cartilage also exhibited significantly more up-regulated VEGF expressions compared to the normal mouse specimens (p < 0.05) (Figure 3B). Moreover, in the IL1-β treated human OA chondrocytes, VEGF expression was significantly increased (Figure 3D) (p < 0.05). These results validate that the angiogenic activator VEGF was up-regulated in pathologic cartilage samples.

### Effect of crizotinib in IL1-β-stimulated chondrocytes

**Figure 4:**
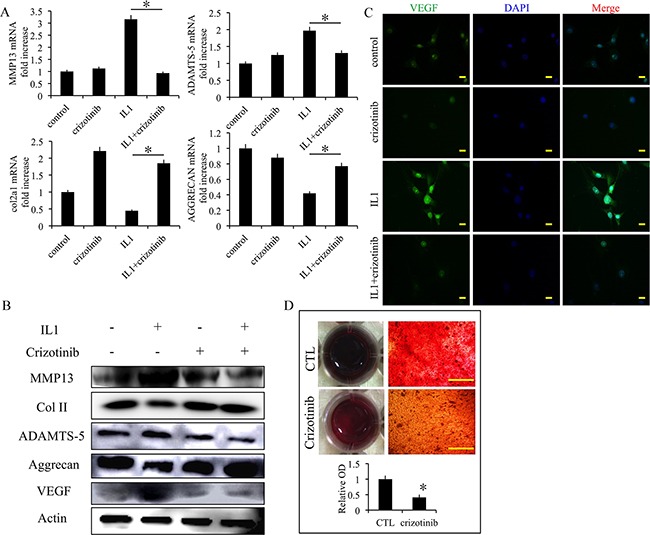
Effect of crizotinib in IL1-β-stimulated chondrocytes **(A)** The effects of crizotinib (10 μM) on mRNA transcript levels of MMP13, COL2A1, ADAMTS-5, and aggrecan after treatment with IL1β (10 ng/mL) for 48 h. n = 3, *p < 0.05 (one way ANOVA). **(B)** Western blot analysis of MMP13, COL2A1, ADAMTS-5, aggrecan, and VEGF protein expression levels after treatment with crizotinib [with or without IL1β (10 ng/mL) stimulation, cropped blots are displayed]. **(C)** Immunocytochemistry detection of VEGF in chondrocytes. Scale bars = 30 μm. **(D)** Effect of crizotinib on chondrocytes calcification visualised by Alizarin Red staining. Scale bars = 30 μm. n = 3, *p < 0.05 (Student's *t*-test).

We next examined the effect of crizotinib in IL1-β-stimulated chondrocytes. The mRNA expression levels of cartilage degradation markers MMP13 and ADAMTS-5 were decreased by crizotinib (10 μM), whereas the expression levels of COL2A1 and aggrecan were increased (Figure 4A). The changes of protein expression levels were similar (Figure 4B). Further, the expression levels of VEGF were assessed. Crizotinib protected from the increase of VEGF in IL1-β-stimulated chondrocytes (Figure 4B). The expression of VEGF was then evaluated by immunofluorescence. The results also exhibited that crizotinib decreased the expression of VEGF in IL1-β-stimulated chondrocytes (Figure 4C). In the chondrocyte calcification model (a model for chondrocyte degeneration), crizotinib decreased formation of calcium nodule in chondrocytes (p < 0.05, Figure 4D). The results suggest that anti-angiogenesis mediated by crizotinib protects IL1-β-stimulated chondrocytes from degeneration and can reduce chondrocyte calcification.

### Effect of crizotinib in cartilage degradation models *ex vivo*

**Figure 5:**
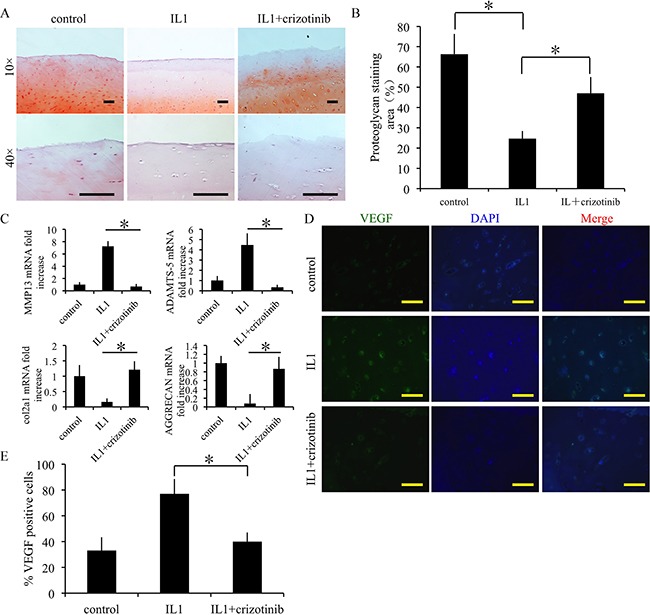
Effect of crizotinib on cartilage matrix degeneration in an *ex vivo* model **(A, B)** Crizotinib significantly attenuated loss of proteoglycan in cartilage induced by IL-1β, assayed by Safranin O staining. Cartilage samples were postmortem from human subjects with no history of OA and cultured with 10 ng/mL IL-1β, in presence or absence of 10 μM crizotinib. Scale bars=100 μm. n = 3, *p < 0.05 (one way ANOVA). **(C)** The effects of crizotinib on mRNA transcript levels of MMP13, COL2A1, ADAMTS-5, and aggrecan. n = 3, *p < 0.05 (one way ANOVA). **(D, E)** Immunocytochemistry was performed to assess expression of VEGF. Scale bars=100 μm. n = 3, *p < 0.05 (one way ANOVA).

The effect of crizotinib was evaluated *ex vivo* in normal cartilage samples from human. IL1-β was used to stimulate cartilage degradation. Crizotinib protected the safranin O-positive proteoglycan in IL1-β-stimulated cartilage from decreasing (p<0.05, Figure 5A, 5B). Further, crizotinib significantly down-regulated MMP13 and ADAMTS-5 mRNA expressions compared to the sample stimulated by IL1-β only (p < 0.05), whereas the expression levels of COL2A1 and aggrecan were up-regulated (Figure 5C). Further, the expression levels of VEGF were assessed by immunofluorescence. Crizotinib significantly down-regulated protein levels of VEGF compared to the sample stimulated by IL1-β only (p < 0.05) (Figure 5D, 5E). The findings suggest that crizotinib may decrease cartilage degenerative changes by inhibition of angiogenesis.

### Effect of crizotinib-loaded CMs combined with GelHA hydrogel *in vivo*

**Figure 6:**
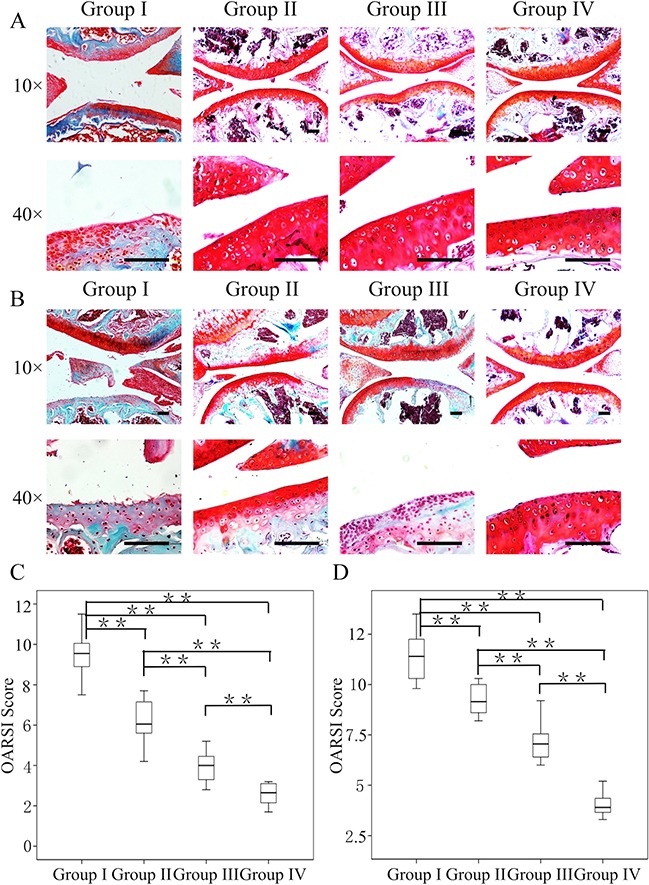
Efficacy of the combination of crizotinib-loaded chitosan microspheres and GelHA hydrogel as a treatment for OA Eight-week-old male C57BL/6 mice were used. Safranin-O staining of cartilage samples 4 **(A)** and 8 weeks **(B)** after OA induction. Scale bars = 100 μm. **(C, D)** OARSI scores of samples 4 and 8 weeks after OA induction. n = 8, *p < 0.05; **p < 0.01 (Kruskal-Wallis ANOVA). Group I, OA mice; group II, OA mice treated with GelHA hydrogel and chitosan microspheres (without crizotinib); group III, OA mice treated with crizotinib alone; group IV, OA mice treated with crizotinib-loaded chitosan microspheres and GelHA hydrogel.

Crizotinib-loaded CMs and GelHA hydrogel were intra-articularly injected to OA joints to delay OA progression. Mice injected with GelHA and CMs (group II) showed marked differences in terms of the OARSI score (p<0.01) at 4 weeks compared to mice untreated (group I). In addition, mice injected with crizotinib alone (group III) exhibited better superficial cartilage and significantly decreased scores compared to the mice in group I (p < 0.01). Notably, OA development was retarded in the mice that received crizotinib-loaded CMs with GelHA hydrogel (group IV), as proved by down-regulated cartilage loss without structural changes (Figure 6A), and the lowest score (2.6 ± 0.6) among all groups (Figure 6C).

At 8 weeks, OA mice untreated exhibited markedly increased cartilage destruction and the highest score among all groups (11.4 ± 1.3, Figure 6B, 6C). Compared to group I, the OARSI scores in groups II and III were significantly lower (p < 0.01). Notably, out of all the experimental groups, group IV had the lowest structural changes and the OARSI score (4.0 ± 0.6) (Figure 6C).

These findings indicate that administering crizotinib alone slowed the progression of surgically induced OA. Furthermore, administering crizotinib with CMs and GelHA hydrogel significantly enhanced the therapeutic effect compared to administering crizotinib alone.

### Effect of crizotinib on cartilage specific degradation markers *in vivo*

**Figure 7:**
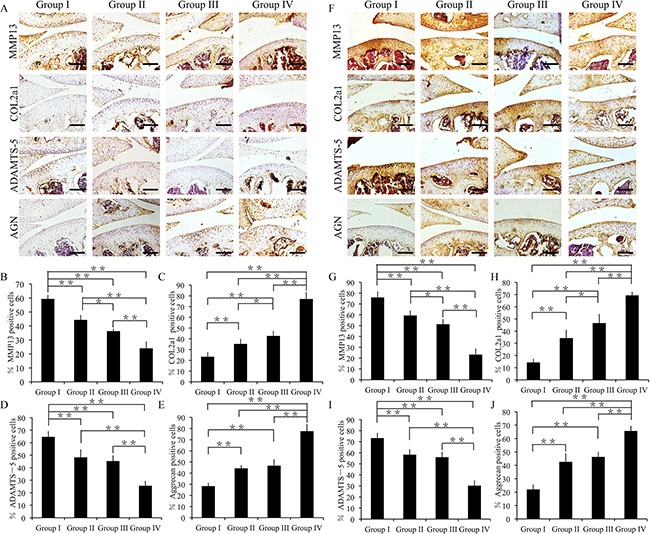
Effect of crizotinib on cartilage matrix degradation *in vivo* **(A)** Immunohistochemistry of MMP13, COL2A1, ADAMTS-5, and aggrecan (4 weeks). Scale bars = 100 μm. **(B, C, D, E)** Quantification of MMP13-positive **(B)**, COL2A1-positive **(C)**, ADAMTS-5-positive **(D)** and aggrecan-positive-cells **(E)** within cartilage samples at 4 weeks post surgery. n = 8, *p < 0.05, **p < 0.01 (one way ANOVA). **(F)** Immunohistochemistry of MMP13, COL2A1, ADAMTS-5, and aggrecan (8 weeks). Scale bars = 100 μm. **(G, H, I, J)** Quantification of MMP13-positive **(G)**, COL2A1-positive **(H)**, ADAMTS-5-positive **(I)** and aggrecan-positive-cells **(J)** within cartilage samples at 8 weeks post surgery. n = 8, *p < 0.05, **p < 0.01 (one way ANOVA).

Immunofluorescence assay was used to evaluate the extent of cartilage degradation. Groups II, III, and IV exhibited significantly lower MMP13 and ADAMTS- 5 expressions compared to group I at 4 weeks (p < 0.01) (Figure 7A, 7B, 7D, Supplementary Figure 2). Moreover, group II showed increased MMP13 expression and reduced COL2A1 expression compared to group III (p < 0.05) (Figure 7A, 7B, 7C). Further, group I showed decreased COL2A1 and aggrecan expressions among all the groups (p < 0.01) (Figure 7A, 7C, 7E).

At 8 weeks, group I exhibited increased MMP13 and ADAMTS-5 expressions compared to groups II–IV (p < 0.01) (Figure 7F, 7G, 7I). In addition, group III showed decreased MMP13 expression and increased COL2A1 expression compared to group II (p < 0.05) (Figure 7F, 7G, 7H). Moreover, group IV exhibited decreased MMP13 and ADAMTS-5 expressions compared to group I-III (p < 0.01) (Figure 7F, 7G, 7I). In particular, groups II, III, and IV exhibited significantly increased COL2A1 and aggrecan expressions compared to group I (p < 0.01) (Figure 7F, 7H, 7J).

Together, these findings suggest that the combination of crizotinib-loaded CMs with GelHA hydrogel can decrease cartilage matrix degradation.

### Effect of crizotinib on VEGF expression *in vivo*

**Figure 8:**
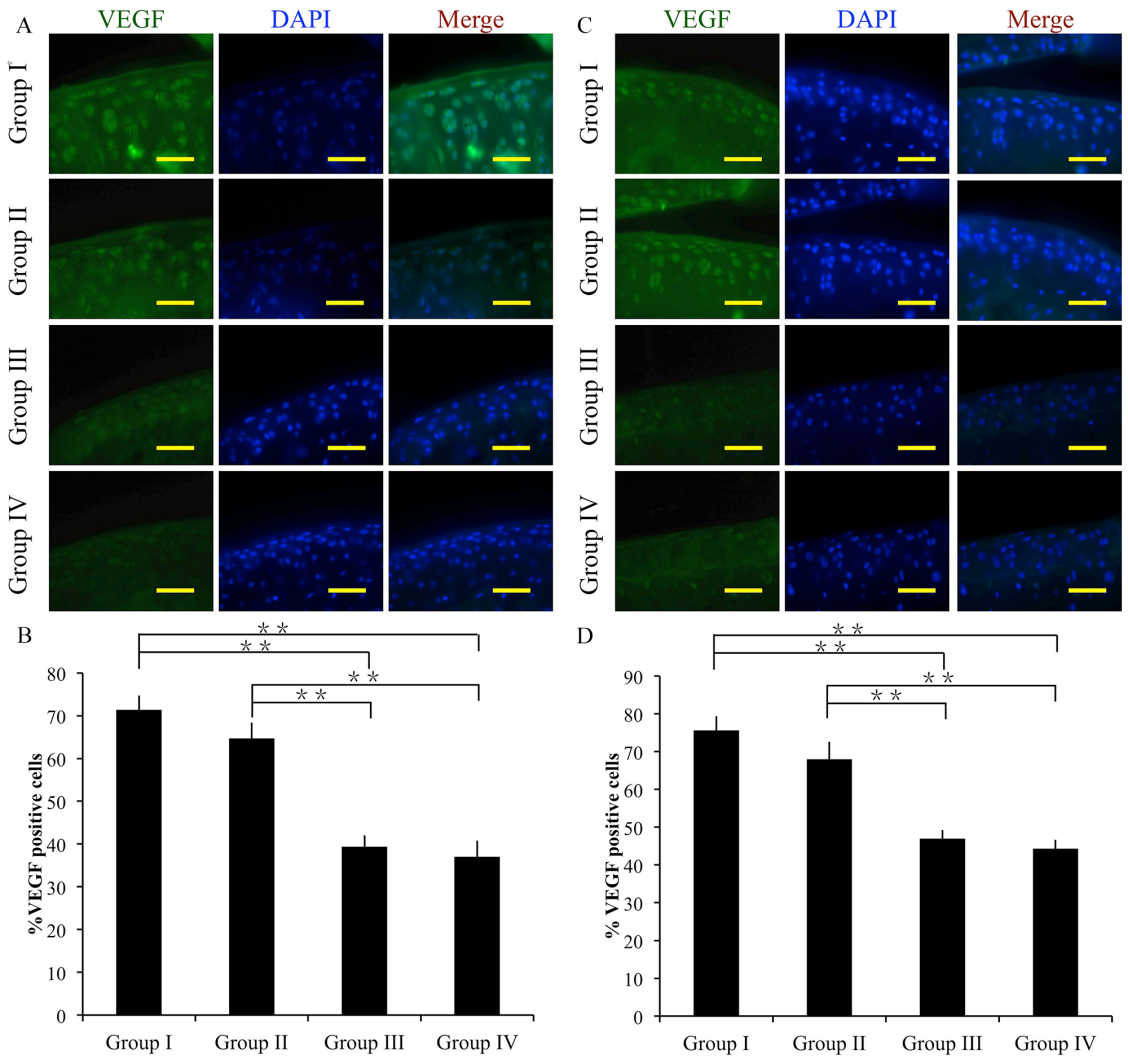
Effect of crizotinib on chondrocyte angiogenesis *in vivo* Immunocytochemistry was performed for VEGF after 4 **(A, B)** or 8 weeks **(C, D)**. Scale bars = 100 μm. n = 8. *p < 0.05, **p < 0.01 (one way ANOVA).

The angiogenic factor VEGF was detected by immunofluorescence. At 4 weeks after surgery, groups III and IV exhibited significantly decreased VEGF expressions compared to group I (p < 0.01). The difference between group I and II was not significant. In addition, group IV exhibited significantly lower VEGF expression compared to group III (p < 0.01) (Figure 8A, 8B).

At 8 weeks, group I showed increased VEGF expression compared to groups III and IV (p < 0.01). However, the difference between group I and II was not significant. And group IV showed significantly lower VEGF expression compared to group III (p < 0.01) (Figure 8C, 8D).

Together, these findings suggest that crizotinib successfully decreases VEGF expression in OA cartilage *in vivo*.

### Mechanism of crizotinib in decreasing cartilage matrix degradation

**Figure 9:**
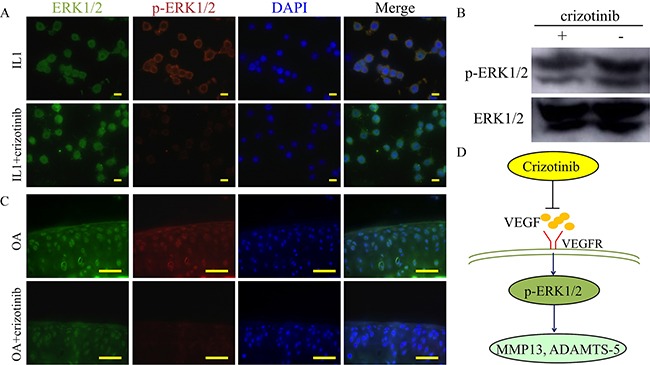
Mechanism of crizotinib in decreasing cartilage matrix degradation **(A)** Immunocytochemistry detection of ERK1/2 and p-ERK1/2 in mouse primary chondrocytes after stimulating with IL1β (10 ng/mL) with or without crizotinib (10 μM) for 1 h. Scale bars = 30 μm. **(B)** Western blot analysis of ERK1/2 signalling in mouse primary chondrocytes after IL1β treatment (cropped blots are displayed). **(C)** Immunocytochemistry was performed for ERK1/2 and p-ERK1/2 in OA mouse cartilage treated with or without crizotinib for 8 weeks. Scale bars = 100 μm. **(D)** A proposed model for the role of crizotinib in osteoarthritis treatment.

To study the mechanisms of how crizotinib decreases cartilage matrix degradation. Phosphorylation status of ERK1/2 (p-ERK1/2) has been regarded as a downstream target of VEGF signaling pathway [22]. And activation of ERK1/2 in articular cartilage causes OA changes such as up-regulation of ADAMTS-5 and MMP13 [23]. As shown in Figure 9A and 9B, p-ERK1/2 expression in mouse chondrocytes was promoted by IL1-β (10 ng/mL) stimulation for 1h. And this effect was impeded by crizotinib (10 μM). Additionally, the phosphorylation of ERK1/2 was observed in mouse OA joints, and this effect was impeded by crizotinib injection (Figure 9C). We therefore hypothesised that crizotinib might reduce the production of VEGF, then impede ERK1/2 phosphorylation and subsequently decrease MMP13, ADAMTS-5 expression.

## DISCUSSION

In this study, we had three major findings. Firstly, angiogenesis was increased in degenerative cartilage as determined by enhanced VEGF expression; secondly, crizotinib ameliorated cartilage degeneration through an anti- angiogenesis function in pathological chondrocytes; and thirdly, the combination of GelHA hydrogel and crizotinib-loaded CMs delayed the OA progression in mice.

Cartilage vascularization is linked with the release of proangiogenic factors such as VEGF and such angiogenesis likely contributes to cartilage structural damage and OA pain [24]. Our study proves that VEGF expression is increased in degenerative cartilage; these results are consistent with previous studies that VEGF is involved in the pathogenesis of osteoarthritis [25]. In addition, it has been shown that an anti-VEGF antibody caused changes of OA-associated protein expressions and ameliorated OA symptoms in animal models [8]. Thus targeting VEGF might achieve a promising therapeutic effect for OA treatment.

Recent research shows that crizotinib can decrease the expression of VEGF [10]. In addition, a patient with an OA diagnosis exhibited apparent improvement in arthritis symptoms when crizotinib was administered. This patient had no OA symptoms by crizotinib treatment [13]. In our experiment, we found that crizotinib can effectively decrease MMP13 and ADAMTS-5 expression [26, 27] in pathological chondrocytes. In addition, crizotinib significantly decreased the expression of the angiogenic factor VEGF *in vitro* and *in vivo*. Thus, we drew the conclusion that crizotinib might ameliorate OA cartilage degeneration via the inhibition of angiogenesis.

The ERK1/2 pathway has been implicated not only in the chondrogenesis but also in growth of endothelial cells [28, 29]. P-ERK1/2 is overexpressed in OA cartilage and subchondral bone tissue, and studies show that by inhibiting of activation ERK1/2 pathway, the progressing of cartilage degeneration is impeded [23]. Our observations proved that crizotinib might decrease expression of p-ERK1/2. Then crizotinib decreased cartilage degradation markers. However, it is not clear how ERK1/2 activation decreases matrix degradation in OA pathogenesis and needs further detailed studies.

In previous studies, it has been shown that GelMA hydrogels have the character of adhesion domains [30], thermosensitivity [31], and enzymatic degradability [32] and support the formation of new extracellular matrix [33]. It has been also been demonstrated that acrylated HA was found to provide a matrix for cell homing and cartilage regeneration [18]. GelHA hydrogels had a lower degradation rate and swelling ratio compared to GelMA hydrogels. In our study, GelHA displayed promising mechanical properties; these findings are consistent with previous studies that hybrid hydrogels exhibit better mechanical properties compared to single components [19]. However, we didn't further investigate the link between the mechanical properties and the therapeutic effects of our hydrogel. This was a limitation of our research. Further work can be done by investigating the association between hydrogel mechanical property and OA therapy. In our *in vivo* experiment, the GelHA hydrogel combined with CMs (without crizotinib) group delayed OA progression. This might be explained by the extracellular matrix properties of GelHA. Hence, GelHA might exert a certain therapeutic effect in OA treatment.

## CONCLUSIONS

In summary, we report that the combination of crizotinib-loaded CMs and GelHA hydrogel delay OA progression. These results suggest that anti-angiogenesis may be a therapeutic approach for OA treatment.

## METHODS

### Materials

Gelatin (Type A, 300 bloom from porcine skin) and methacrylic anhydride (MA) were purchased from Sigma-Aldrich (St. Louis, MO, USA). Sodium hyaluronate was purchased from Aladdin Chemistry Co., Ltd. (Shanghai, China). All other chemicals were purchased from Sigma-Aldrich unless specifically mentioned.

### Synthesis of polymer precursors

GelMA was synthesized according to a procedure described previously [34]. Type A porcine skin gelatin was mixed into phosphate-buffered saline (PBS) at 60°C and stirred until fully dissolved at a concentration of 10% (w/v). MA was added to the gelatin solution at a rate of 0.5 mL/min until the target volume was reached, stirred at 50°C, and allowed to react for 1 h. Following a 5× dilution with additional warm (40°C) PBS to stop the reaction, the mixture was dialyzed against distilled water using 12–14 kDa cutoff dialysis tubing for 1 week at 40°C to remove salts and methacrylic acid. The solution was lyophilized for 1 week to generate a white porous foam and stored at −80°C. HAMA was also synthesized following a previously described procedure [35]. Briefly, 1 g hyaluronic acid sodium salt was dissolved in 100 mL distilled water until it fully dissolved. MA was then added to this solution at 1% (v/v) and the reaction was performed for 24 h at 4°C by maintaining the pH between 8–10 with the addition of 5 M sodium hydroxide. The resulting solution was dialyzed in a 12–14 kDa dialysis membrane at 4°C for 3 days, frozen at −80°C, and freeze dried to obtain a solid product, which was then kept at −80°C until further use.

### Production of hybrid hydrogels

The prepolymer hydrogel solution was prepared by mixing 5 wt% GelMA solution and 2 wt% HAMA into PBS containing 1% (w/v) 2-hydroxy-1- (4-(hydroxyethoxy)phenyl)-2-methyl-1-propanone (Irgacure 2959, CIBA Chemicals, Basel, Switzerland) as a photoinitiator at 80°C. The prepolymer solution was vigorously stirred at room temperature for 10 min to generate a homogeneous solution, which was pipetted into a 24-well culture dish (200 μL/well) and exposed to UV light (320–500 nm, 7.0 mW/cm^2^) for 2 min to allow for photo-crosslinking. The samples were incubated in a free-floating manner at 37°C in PBS for 24 h, followed by storage at −20°C.

### Characterization of the hydrogels

The hydrogel swell ratio was analyzed as follows. The hydrogels were lyophilized until dry and dry weight (Wd) was measured. Dried hydrogel samples (n = 3) were immersed in 50 mL PBS at 37°C and allowed to swell. Swollen hydrogel samples were weighed to determine swollen weight (Ws) at different time points. The swelling ratio (Q) was calculated by the following equation: Q = Ws/Wd.

To characterize the enzymatic degradation properties of the hydrogel, we placed the hydrogel samples in 1.5-mL centrifuge tubes with 1 mL PBS containing 1–2 U/mL collagenase type II at 37°C. At a pre-defined time, the hydrogels for each condition were removed, frozen, and lyophilized. Mass loss was determined as the ratio of the final weight to the original dry weight. All experiments were repeated 3 times.

### Mechanical testing

The hydrogels for mechanical testing were produced as described in section 2.4. After UV crosslinking, the hydrogels were rinsed with PBS and further maintained in PBS for 24 h. The hydrogels were punched using an eight mm biopsy punch prior to mechanical testing. The excess liquid from the hydrogel disks was removed using Kimwipes. Compression testing was carried out by applying a strain rate of 0.2 mm/min using an Instron testing machine (model 5543; Instron, Canton, MA, USA) and software (Bluehill V2.0; Instron). We determined the compressive modulus by taking the slope in the linear section of the stress-strain curve at 5%–10% strain area. Three replicates were used for each hydrogel composition.

### Preparation of chitosan microspheres

Chitosan microspheres were prepared through the water-in-oil (W/O) emulsion solvent diffusion method as previously described [34]. CMs were prepared using the water-in-oil (W/O) emulsion solvent diffusion method. Chitosan solution (2% w/v) was prepared by dissolving chitosan (Shanghai Bio Science and Technology) in 2.5% (v/v) acetic acid aqueous solution (Sinopharm Chemical Reagent Co., Ltd.) at room temperature. The chitosan solution was mixed with Pal by stirring overnight with a magnetic stirrer to produce a homogeneous mixture. Next, 5 mL of the resulting mixture was aspirated into a syringe pump and added drop-wise into the oil phase (24.72 mL), which consisted of 14 mL of liquid paraffin (Sinopharm Chemical Reagent Co., Ltd.), 10 mL of petroleum ether, and 0.72 mL of Span 80 (Sangon Biotech), at a flow rate of 4 mL/h with continuous stirring at 1500 rpm. A syringe needle with an internal diameter of 0.2 mm was used for this process. After the solvent diffusion procedure, the suspension was cross-linked using 25% (v/v) glutaraldehyde solution as a cross-linking agent. The addition of the cross-linker was performed three times, at time intervals of 15 min, with the following volumes of glutaraldehyde: 0.64, 0.64, and 0.32 mL. Subsequently, the suspension was stirred at room temperature to produce cross-linking and centrifuged at 3000 rpm for 5 min, after which the supernatant fluid was discarded. Next, the microspheres were washed with petroleum ether (three times) (Sinopharm Chemical Reagent Co., Ltd.), methanol (two times) (Sinopharm Chemical Reagent Co., Ltd.), acetone (one time) (Sinopharm Chemical Reagent Co., Ltd.), isopropyl alcohol (one time) (Sinopharm Chemical Reagent Co., Ltd.), ethanol (one time), and distilled water (three times). Once the washing procedure was complete, the microspheres were collected after lyophilization with a freeze-dryer to remove residual water. For the control group, pure CMs were prepared by directly dropping the chitosan solution into the oil phase under the same conditions.

### *In vitro* crizotinib release profile

To evaluate the absorption peak of crizotinib, the UV-vis spectrum from 200 to 1000 nm at step size of 2 nm was obtained using a Beckman DU640UV-vis spectrophotometer (Beckman-Coulter, Brea, CA, USA). The absorption curve showed that the drug had absorption peaks at 265 and 317 nm. To characterize the standard curve of crizotinib, a suitable amount of crizotinib was weighed to prepare different concentrations of crizotinib solutions (5, 10, 20, 40, 80, and 160 mg/L). The optical density (OD) values of the crizotinib solution samples were quantified using the spectrophotometer at 265 nm. To ensure that crizotinib was encapsulated into CMs, the prepared crizotinib-loaded CMs were characterized by FTIR spectroscopy (Nicolet AVA TAR370, Thermo Scientific, Waltham, MA, USA). The *in vitro* crizotinib release profile from the crizotinib-loaded CMs and crizotinib-loaded CMs with hydrogel were determined at pH 7.4 with 0.01 M PBS solution containing 0.5% (v/v) dimethyl sulphoxide (DMSO). The amount of crizotinib, CMs and hydrogel was 0.4 mg, 100 mg, and 40 mL, respectively. The crizotinib-loaded CMs with or without hydrogel were placed in a 50 mL Eppendorf tube. Then, 20 mL 0.01 M PBS was added into the tube. The sample was incubated at 37°C with gentle agitation. At the desired times (0, 1, 3, 6, 12, 18, 24, 36, 48, 60, 72, 84, and 96 h), the OD values of 100 mL solutions were quantified using a spectrophotometer at 265 nm. At each time point, the detected solutions were pipetted back into the sample to ensure a total volume of 20 mL. The experiments were carried out in triplicate.

### Scanning electron microscopy characterization

To characterize the internal microstructures of the materials, the samples were frozen at −80°C and lyophilized. The dried samples were mounted on aluminum stubs, sputter-coated with gold, and observed under a scanning electron microscope (Hitachi S3000N) at an accelerating voltage of 15 kV.

### Human cartilage, primary cultures of human chondrocytes and primary cultures of mouse chondrocytes

These methods were carried out in accordance with human tissue samples' guidelines and regulations. And the experimental protocols were approved by the Ethics Committee of Sir Run Run Shaw Hospital. The patients' informed consent was obtained from all subjects prior to harvesting of human tissue samples. Control normal cartilage was obtained postmortem from human cadavers with no history of OA. Pathological cartilage was obtained from OA patients undergoing total knee replacement surgery. Human articular chondrocytes were harvested by overnight incubation of 1 mm^2^ cartilage slices with 2 mg/mL of collagenase P in Dulbecco's modified Eagle medium (DMEM) supplemented with 10% fetal bovine serum and 40 μg/mL gentamicin at 37°C. After resuspension and filtration through a 0.7 μm filter, cells were cultured in a 24-well plate at a seeding density of 2×10^5^ cells/mL.

Mouse articular cartilage was obtained from the femoral condyles and tibial plateaus of C57BL/6 mice on postnatal day 5–6, as described previously [36]. Chondrocytes were maintained as a monolayer in DMEM supplemented with 10% (v/v) fetal bovine serum at 37°C. Cells were utilized in the experiments through the 3rd passage.

### Cartilage explant cultures

The explants were initially cultured at 37°C with 5% CO_2_ in 10% FBS (V/V), antibiotics, and 2 mM L-glutamine (Invitrogen). After 2 days, the explants were washed in serum-free medium and placed in a 48-well culture dish with fresh serum-free medium containing 10 ng/ml human recombinant interleukin-1(IL-1) or 10 ng/ml IL-1 with 10 μM crizotinib and cultured for a further 3 days. The explants were collected on days 3 after changing to serum-free medium.

### Quantitative Real-Time Polymerase Chain Reaction

Explants were washed twice with PBS and physically crushed using a Cryo-Press CP-100WP (Microtech Nichion, Chiba, Japan). Crushed explants were harvested and total RNA was extracted from these cells using TRIzol (Invitrogen Inc., Carlsbad, CA, USA). Total cellular RNA from the chondrocytes was also extracted by Trizol according to the manufacturer's instructions. PCR was performed using SYBR Green QPCR Master Mix (Takara) with a Light Cycler apparatus (Bio-Rad, CFX-Touch). The PCR cycling consisted of 40 cycles of amplification of the template DNA with an annealing temperature of 60°C. The relative level of expression of each target gene was calculated using the 2-ΔΔCt method. The amplification efficiencies of each primer pair were validated to enable quantitative comparison of gene expression. Each real-time PCR was performed on at least 3 different experimental samples. Representative results are displayed as target gene expression normalized to the reference gene actin. Error bars represent one SD from the mean of technical replicates. All primer sequences (Invitrogen Inc., Carlsbad, CA, USA) were designed using primer 5.0 software. The following primer sequences were utilized: 5′-C GAGTGGAAGAGCGGAGACT-3′ and 5′-AACTTTCATG GCGTCCAAGGT-3′ (mouse COL2A1); 5′-GAAGAGCCTCGAATCACCTG-3′ and 5′-ATCCTGGGCACATTA TGGAA-3′ (mouse Acan); 5′-ATCCAGCTAAGACACAGCAAGCCA-3′ and 5′-TGGAGCACAAAGGAGTGGTCTCAA-3′ (mouse MMP13); 5′-GC TACTGCACAGGGAAGAGG-3′ and 5′-TGCATATTTGGAACCCATT-3′ (mouse ADAMTS-5); 5′-CTGGAAAAGCTGGTGAAA GG -3′ and 5′- GGCCTGGATAACCTCTGTGA-3′ (human COL2A1); 5′- TCCCCTGCTATTTCATCGAC-3′ and 5′-CCAGCAGCACTA CCTCCTTC-3′ (human Acan); 5′-AACATCCAAAAACGCCAGAC -3′ and 5′-GGAAGTTCT GGCCAAAATGA-3′ (human MMP13); 5′-TACTTGGCCTC TCCCATGAC-3′ and 5′- TTTGGACCAGGGCTTAGATG -3′ (human ADAMTS-5).

### Western blot analysis of protein expression

Protein expression levels of VEGF (Abcam, ab46154), MMP13 (Santa Cruz, sc30073), COL2A1 (Abcam, ab116242), ADAMTS-5 (Santa Cruz, sc83186), aggrecan (Santa Cruz, sc25674), ERK1/2 (Abcam, ab17942) and p-ERK (Santa Cruz, sc7383) were measured by western blotting. Cartilage samples were first dissected into small pieces (0.5 mm×0.5 mm) with a knife, and then milled in 200 μL radioimmune precipitation assay (RIPA) lysis buffer with protease inhibitor cocktail and phenylmethanesulfonyl fluoride with a homogeniser (Ultra-Turrax IKA T10 basic). The mixture was then centrifuged at 10 000 g, with the supernatant being collected and used for VEGF activity assay. Cytosolic proteins from chondrocytes were directly extracted with RIPA lysis buffer containing a protease inhibitor cocktail. The extracted proteins were separated on SDS-PAGE gels. After electrophoresis, the separated proteins were transferred onto polyvinylidene difluoride membranes and blocked in 5% (w/v) non-fat milk for 4 h at room temperature. Each membrane was incubated overnight at 4°C with the appropriate primary antibodies and washed in Tris-buffered saline with Tween (TBST). Next, the membranes were incubated in horseradish peroxidase (HRP)-conjugated secondary antibodies (diluted 1:1500 in 5% (w/v) bovine serum albumin (BSA) solution) for 2 h at room temperature. Excess secondary antibodies were rinsed off the membrane with TBST, after which a chemiluminescent signal was generated using western blot detection reagents (ECL, Beyotime Institute of Biotechnology) according to the manufacturer's protocol.

### Chondrocyte calcification *in vitro*

Cells were trypsinised and seeded into a 24-wells plate, and calcification was induced for 2 weeks with calcification medium, composed of DMEM supplemented with 1% ITS+ (BD Biosciences, Franklin Lakes, New Jersey, USA), 1% antibiotic-antimycotic solution, 50 μg/mL ascorbate-2-phosphate (Sigma, St Louis, Missouri, USA), 40 μg/mL L-proline (Sigma), 100 nM dexamethasone (Sigma) and 1 nM triiodothyronine (T3) (Sigma).

### OA-inducing anterior cruciate ligament transection (ACLT) surgery and intra-articular delivery of crizotinib

All experiments involving mice were performed with the approval of the Zhejiang University Ethics Committee. Eight-week-old male C57BL/6 mice were administered ACLT (anterior cruciate ligament transection) surgery to both knees as described previously [37]. Immediately after surgery, the animals were returned to their individual cages without joint immobilization. The mice in group I was left untreated. Ten days after ACLT, intra-articular injections of GelHA with CMs (10 μl, group II), crizotinib alone (3.5 mg/kg, group III), crizotinib-loaded CMs (crizotinib concentration: 3.5 mg/kg) combined with GelHA hydrogel (10 μl, group IV) were administered to the mice twice per week. The intra-articular injection was performed as previously described [38]. With the mouse in a supine position, the knee was placed into a custom made holder such that the fixation screws were aligned just above the patella tendon. The screws were tightened superior to the lateral and medial epicondyles to immobilize the femur. A small skin incision was made to expose the patella and the tendon. Using the micromanipulator, a 30G needle was inserted through the tendon and into the space between the patella and the femur. Entry into the joint space was considered successful when the patella could be seen lifting upwards as the needle slid underneath. Each group consisted of eight mice. The mice were sacrificed 4 and 8 weeks after ACLT. The distal femur of each mouse was dissected, embedded in paraffin, and investigated by safranin-O staining and immunostaining.

### OARSI scoring of murine cartilage

Semi-quantitative histopathological grading was performed using a modified version of the Chambers scoring system [39–41], which has been established by the OARSI histopathology initiative as the standard method for grading mouse cartilage degeneration [42]. Based on this system, paraffin sections from each sample were scored after safranin-O staining. Histological grading was performed in 4 areas: medial femoral condyle, medial tibial plateau, lateral femoral condyle, and lateral tibial plateau. The grades of three blinded observers for each section were averaged, after which the data from each group of mice were collated.

### Histological analysis, immunohistochemistry and immunofluorescence of human cartilage murine knee joints and mouse chondrocytes

The isolated knee joints were processed for histology and immunohistochemistry. Tissue samples were fixed in 4% (v/v) neutral buffered formalin for 24 h and decalcified in neutral 10% EDTA solution for 1 month at room temperature. Subsequently, the samples were dehydrated in an alcohol gradient, cleared, and embedded in paraffin blocks. Histological sections (8 μm) were prepared using a microtome. Six representative sections of each joint from various depths were mounted on slides, stained with safranin-O, and photographed digitally under a microscope. Human cartilage sections (8 μm) were prepared using a freezing microtome. After overnight incubation at 4°C with primary antibodies for MMP13, COL2A1, ADAMTS-5, and aggrecan, the histological sections were incubated with secondary antibodies (Beyotime Institute of Biotechnology Inc., Jiangsu, China) for 2 h at room temperature. The DAB substrate system (Zsbio, Beijing, China) was used for color development. Hematoxylin staining was utilized to reveal the nuclei of the cells.

Mouse sections or chondrocytes evaluated by immunofluorescence were blocked by incubation with 5% (w/v) BSA, incubated with primary antibodies for VEGF, ERK1/2 or p-ERK, incubated with corresponding secondary antibodies conjugated to Alexa Fluor 488 or Alexa Fluor 546 fluorescent dye (Invitrogen), and stained with DAPI (Beyotime Institute of Biotechnology). After staining, the histological sections were viewed under a microscope. The number of positively stained cells on the entire articular surface (including the femoral condyle and tibial plateau area) per specimen was counted in five sequential sections per joint in each group [43].

### Statistical analysis

All quantitative data are presented as mean ± SD. VEGF expression in normal and OA cartilage samples from humans or mice, VEGF expression in human OA chondrocytes treated with or without IL1β and OD values of Alizarin Red staining were compared using the Student's *t*-test. Histological grades are non-parametric ordinal ranks, and thus the OARSI scores among different groups were compared using Kruskal-Wallis analysis with the Mann-Whitney U test. Other statistical data were analyzed using one-way ANOVA/ Tukey's post-hoc test. For all experiments, P < 0.05 was considered to be significant and is indicated by *. P < 0.01 is indicated by **.

## SUPPLEMENTARY FIGURES


