# Supplementary material for: The amelioration of cartilage degeneration by photo-crosslinked GelHA hydrogel and crizotinib encapsulated chitosan microspheres
Source: Oncotarget. 2017 Feb 27;8(18):30235–51. doi: 10.18632/oncotarget.15750 (PMC5444739; doi:10.18632/oncotarget.15750)
Supplement: Supplementary file 1 [file oncotarget-08-30235-s001.pdf]

The amelioration of cartilage degeneration by photo-crosslinked GelHA hydrogel and crizotinib encapsulated chitosan microspheres

SUPPLEMENTARY FIGURES

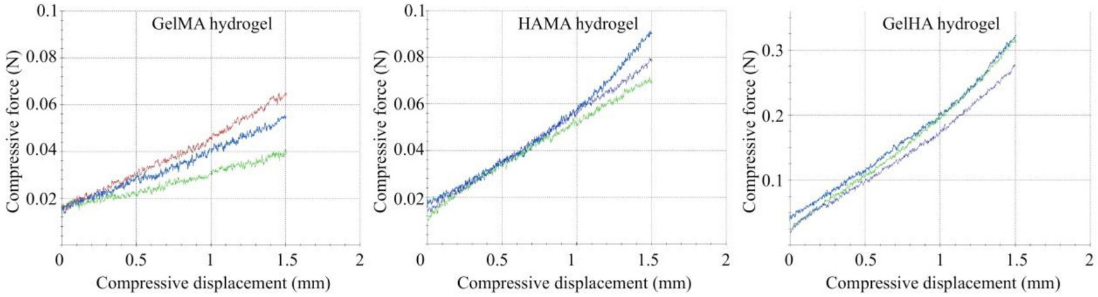

Supplementary Figure 1: Load-displacement curves of the hydrogels.

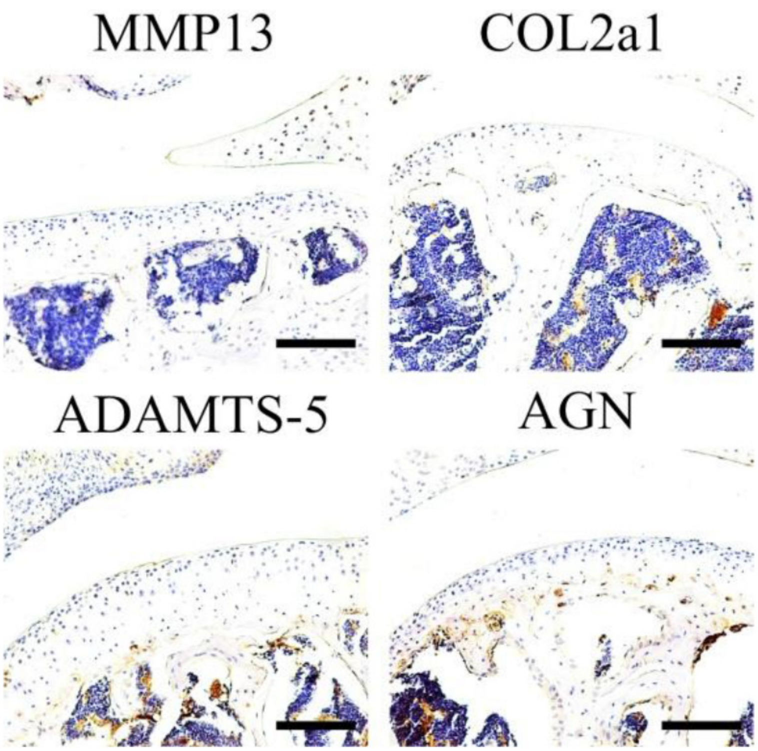

Supplementary Figure 2: Negative controls of MMP-13, COL2A1, ADAMTS-5, aggrecan. Scale bars = 100 µm.
